# Clinical efficacy and safety of nuanxin capsule for chronic heart failure: A systematic review and meta-analysis

**DOI:** 10.1097/MD.0000000000011339

**Published:** 2018-07-06

**Authors:** Ziqing Li, Yu Zhang, Tie Yuan

**Affiliations:** a The Second Clinical School of Guangzhou University of Chinese Medicine; b Guangdong Provincial Hospital of Chinese Medicine, The Second Clinical School of Guangzhou University of Chinese Medicine, Guangzhou, China.

**Keywords:** chronic heart failure, nuanxin capsule, protocol, systematic review

## Abstract

**Background:**

Chronic heart failure (CHF), the final phase of various heart diseases, is a serious public health problem resulting in high hospitalization rates, mortality, and increasing health care costs. Nuanxin capsule (NXC), a Chinese herbal formula, has been widely used in the treatment of CHF. However, the safety and efficacy of NXC used in patients with CHF has been uncertain and there has been no standard clinical trial published to confirm this. Thus, we conduct a study to evaluate the safety and efficacy of NXC for CHF.

**Methods:**

The reference lists of randomized controlled trials and 8 electronic databases will be independently and systematically searched by 2 review authors in May 2018. Four English databases (EMBASE, PubMed, Cumulative Index to Nursing and Allied Health Literature [CINAHL], and Cochrane Central Register of Controlled Trials [CENTRAL]) and 4 Chinese databases (Chinese Biomedical Literature Database [CBM], Chinese National Knowledge Infrastructure [CNKI], Wanfang Database, and VIP Database) will be included. The primary outcomes will be assessed according to the function classification of New York Heart Association (NYHA). Data synthesis will be precisely computed using the RevManV5.3 software when a data-analysis is allowed. Methodological quality will be assessed according to Cochrane Handbook.

**Results:**

This study will provide a high-quality synthesis of current evidence of NXC for CHF from different aspects including the mortality, the function classification of NYHA.

**Conclusion:**

The conclusion of this systematic review will provide evidence to prove whether NXC is an effective therapeutic intervention for patient with CHF.

PROSPERO registration number: PROSPERO CRD42018090003.

## Abstract 2

Supplemental Digital Content is available in the text

## 1 Introduction

Chronic heart failure (CHF), a major public health issue, is the final stage of various cardiovascular diseases, which occurs when the blood cannot be pumped within the circulatory system and the oxygen and nutrients cannot be sent to the organs.^[1]^ Epidemiological studies have revealed that in western countries, the prevalence rate of CHF is about 1% to 2%, which means almost every 5 to 10 in 1000 people will be diagnosed with CHF per year.^[2]^ The evidence from American Heart Association's study has showed that the 2013 overall mortality rate attributable to heart disease was 222.9 per 100,000 Americans.^[3]^ It is >650,000 new CHF patients that are added annually in the United States.^[4]^ During 2012, the cost of CHF in China was up to $5.42 billion according to the World Bank Asian Development Indicators, which ranked as the greatest healthcare burden among low and middle-income countries.^[5]^ Not only the rate of death, hospitalization, and rehospitalization, but also the serious impacts on the quality of patients’ life are caused by this disease.^[6,7]^ The traditional treatment methods for CHF involves vasodilators, diuretics, and antihypertensive.^[8–10]^ According to a plenty of randomized controlled trails (RCTS), it has been reported that β-blockers, aldosterone antagonists, angiotensin-converting enzyme inhibitors (ACEI), and angiotensin receptor blockers (ARB) can slow down the progression of myocardial reconstruction and the development of CHF.^[11]^ Meanwhile, despite the alleviation and advance of symptoms in the treatment for CHF, there are still adverse events that limit the use of the traditional drugs. For example, when patients take the antihypertensive drugs, some common adverse effects such as headache and bradycardia will frequently occur.^[12,13]^

Furthermore, the recent data from a study of congestive heart failure prospective cohort has demonstrated that the mortality overall within a year was 16.5%.^[14]^

Chinese herbal medicine (CHM), a supplementary therapy originated in ancient China and based on traditional Chinese medicine (TCM), has been widely used in the treatment of cardiovascular diseases, such as CHF.^[15]^ TCM plays a significant role in treating CHF in China with a lower cost and a distinct therapeutic effect on CHF-related symptoms.^[16]^ Nuanxin capsule (NXC), a modern Chinese medical preparation, is derived from TCM prescription and modified by Deng Tietao, a famous veteran doctors of TCM, who puts forward a set of proposals that the primary cause of CHF is heart Qi deficiency and blood stasis. NXC has a composition of 5 Chinese herbs, including Radix Ginseng, radix aconite lateralis preparata, pseudo-ginseng, coix seed, and epicarpium citri.^[17]^ Several animal experimental studies have demonstrated that NXC has good therapeutic effects on cardiac function in rat models of CHF.^[18,19]^ Many RCTs have reported that using NSC together with western medicine can be more effective than that of just using western medicine for patient with CHF.^[20,21]^ However, to our knowledge, there has been no standard clinical study published to prove the efficacy and safety of NXC in treating patients with CHF. Therefore, the main objective of this systematic study is to assess the efficacy and safety of NXC in the treatment of CHF.

## 2 Methods

### 2.1 Inclusion criteria for study selection

#### 2.1.1 Types of studies

All the RCTs that investigated the effect of NXC for the treatment of CHF will be included. Nonrandomized clinical studies and case studies will be excluded. No publication type of restriction or writing language will be applied in this study.

#### 2.1.2 Types of patients

Trials involving participants with CHF will be included without limitations of age, sex, education status, or ethnic background. Patients with CHF should be diagnosed by physicians based on the diagnostic standard established by the New York Heart Association (NYHA).^[22]^

#### 2.1.3 Types of interventions

According to the treatment guidelines of heart failure, the participants in the experimental group will be treated with the therapeutic intervention of conventional western medications and NXC, whereas the controlled group will be treated with the conventional western medications.

#### 2.1.4 Types of outcome measures

##### 2.1.4.1 Primary outcomes

Primary outcomes are:

- Composite cardiac events (CCEs)
- Left ventricular ejection fraction (LVEF)
- NYHA functional classification

##### 2.1.4.2 Secondary outcomes

Secondary outcomes are:

- Exercise test or 6-minute walking distance (6MWD).
- Adverse events.
- Patient quality of life according to the Chronic Heart Failure Quality of Life Scale of Integrated Chinese and Western Medicine.^[23]^

### 2.2 Search methods for the identification of studies

#### 2.2.1 Electronic searches

Two research members (YW and YZ) will electronically and independently search four English databases (EMBASE, PubMed, Cumulative Index to Nursing and Allied Health Literature [CINAHL], and Cochrane Central Register of Controlled Trials [CENTRAL]) and 4 Chinese databases (Chinese Biomedical Literature Database [CBM], Chinese National Knowledge Infrastructure [CNKI], Wanfang Database and VIP Database) from their inception to May 2018. The searched items will be used as follows: CHF, NXC, and RCTs. The same terms will be searched in the Chinese databases. The data will be retrieved with the combination of medical keywords and uncontrolled terms. The detailed retrieval strategy of PubMed database will be shown in Appendix A and will be constantly modified by searching other databases.

#### 2.2.2 Searching other resources

Meta-analysis of RCTs and recently relevant systematic reviews will be electronically searched. Furthermore, the related conference proceedings and reference list of eligible studies will be particularly searched to avoid the eligible trials as well.

### 2.3 Data collection and analysis

#### 2.3.1 Selection of studies

**Figure 1:**
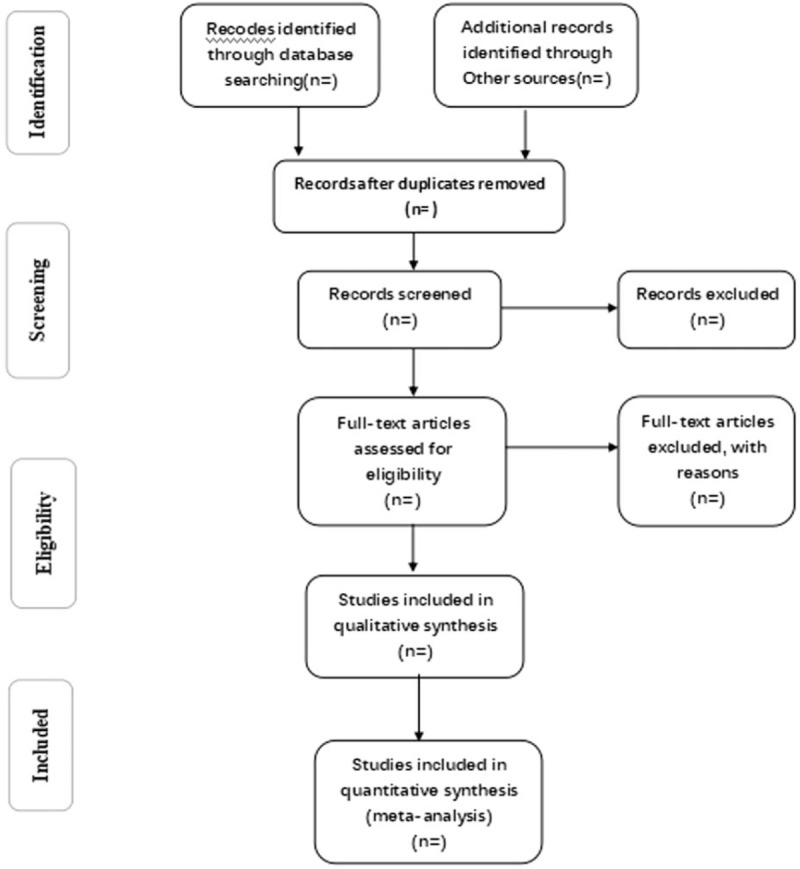
Flow diagram of study selection process.

Reviewers will receive professional training to be familiar with the background, objective, and process of this review. Relevant studies obtained from the databases will be uploaded to a literature management system of EndnoteX8. Two search reviewers will independently carry out the selection and record their decisions with a standard eligibility form by screening the titles and abstracts of the retrieved articles. They will read the whole article later to meet the requirements and check the final inclusion of references. Any disagreement between 2 reviewers about the inclusion of studies will be resolved through discussion. If the discussion cannot reach a consensus, the third search reviewer will make a final decision of the selection. Details of the selection process of studies will be shown in a PRISMA flow chart (Fig. 1).

#### 2.3.2 Data collection and management

Two search reviewers will read all the included articles and independently collect data via a standardized eligibility form. General information of the retrieved articles will be extracted, including the first author, year of publication, study design, sample size, age, sex, duration and severity of disease, intervention, and treatment applied in the control group. Outcome measures and further information such as results of the study, adverse events, and conflicts of interest will be extracted as well. Any divergence of the data extraction will be discussed and resolved between 2 search reviewers. The final results of the data extraction will be collated by the arbiter. When the data are insufficient or ambiguous, one of the reviewers will contact the original author to acquire additional and detailed information by telephone or e-mail.

#### 2.3.3 Assessment of risk of bias in included studies

Two review authors will independently assess the risk of bias of the included studies according to the Cochrane Collaboration's tool provided by Cochrane Handbook V.5.1.0, which involves the following 7 domains: blinding of participants, randomized sequence generation, allocation concealment, personnel and outcome assessors, selective reporting, incomplete outcome data addressed, and other issue. The result of the evaluated domains will be classified into 3 levers: low risk, high risk, and unclear risk. Any discrepancies will be resolved through team discussion to reach an agreement. If necessary, a third reviewer will be consulted to confirm the details.

#### 2.3.4 Measures of treatment effect

For dichotomous outcomes, a relative risk (RR) with 95% confidence interval (CI) will be used to identify the effect of treatment. For continuous and integrated data, a mean difference (MD) will be presented with 95% CI to evaluate the extracted data.

#### 2.3.5 Unit of analysis issue

Only the data obtained from the meta-analysis and RCTs studies will be adopted. If the data of crossover trial are involved, we will merely use the first-phase data. If the unit of analysis has multiple time points to observe, we will divide the time point observation into 2 terms: a short term (within 1 month) and a long term (over 1 month).

#### 2.3.6 Dealing with missing data

If it is possible, we will try to come in contact with the first author via e-mail or telephone to request for the inadequate and missing data. If it cannot work, the analysis will be built only with the available data and potential effect of the missing data.

#### 2.3.7 Assessment of heterogeneity

According to the guideline of Cochrane Handbook V.5.1.0, the heterogeneity of the results can be evaluated with the *χ*^2^ test (a = 0.1). If the value determined by *I*^2^ exceeds 50%, the heterogeneity among trials will be considered to be significant. Subgroup analysis will be carried out to explore the correlative causes of heterogeneity.

#### 2.3.8 Assessment of reporting biases

If sufficient trials are included in the study (>10 trials), visual asymmetry on a funnel plot will be utilized to detect reporting bias and the funnel plot asymmetry will be evaluated with the use of the test of Egger regression.

#### 2.3.9 Data synthesis

RevMan software V5.3 from Cochrane Collaboration will be used to compute the data synthesis and conduct meta-analysis when suitable. The analysis with a fixed-effect model will be performed to calculate the RR and MD with low heterogeneity (*I*^2^ < 50%). If not, a random-effect model will be employed to synthesize the data.

#### 2.3.10 Subgroup analysis

Owing to the inconsistency among participant characteristic, detailed interventions, and outcome measures, subgroup analysis will be performed if the amount of included trials is sufficient (at least 10 trials). Subgroup analysis is carried out to explore the potential causes of the heterogeneity.

#### 2.3.11 Sensitivity analysis

We will perform sensitivity analysis to determine the quality and robustness of results according to the following criteria: sample size; analysis issue (such as the impact of missing data); methodological quality.

#### 2.3.12 Dissemination and ethics

The results of this systemic review will indicate the efficacy and safety of NXC for CHF. This review will be disseminated through publication in a peer-reviewed journal and presentation at a relevant conference. It is not necessary for a formal ethical approval because the data are not individualized.

#### 2.3.13 Grading the quality of evidence (Summary of evidence)

The Grading of Recommendations Assessment, Development and Evaluation (GRADE) is a tool to evaluate the quality of primary outcome. The level of evaluation will be divided into 4 types: high, moderate, low, or very low.

## 3 Discussion

CHF is a major health issue, the incidence of which occurs worldwide. Although the medical technology improves rapidly in the treatment of CHF, the mortality and morbidity of CHF are still increasing year by year.^[14]^ During the development of CHF, myocardial fibrosis is of crucial importance, especially the angiotensin II and aldosterone of the renin-angiotensin-aldosterone system (RAAS), which play an important role in myocardial fibrosis.^[24,25]^ A published study has reported that the activation of the RAAS and sympathetic nervous system results in cardiac hypertrophy, which may lead to ventricular remodeling and ultimately decompensation.^[26]^ Recent studies have discovered substantial evidence of the limitation in the drugs, such as ACEIs and ARBs.^[27,28]^

To deal with that, TCM has been widely applied in treating CHF in China with its better therapy efficacy, fewer side effects and lower cost on CHF. NXC, a modern prescription originated from TCM, has been put into use in the treatment of CHF and has effective symptom improvement in clinical practice. Several published studies have demonstrated that NXC could have an effective impact on the treatment of CHF with alleviation of symptoms, such as heart palpitation and chest pain.^[21,22]^ However, relevant evidence from clinical studies and the exact mechanism of NXC in treating CHF still need further exploration. In addition, there has been no meta-analysis or systematical review published to prove the efficacy and safety of NXC in the treatment of CHF in English. Therefore, the objective of this systematic study is to evaluate the efficacy and safety of NXC in CHF and this study will try to provide more high-quality medical evidence on the safety and efficacy of NXC in the treatment of CHF.

## Author contributions

**Conceptualization:** Ziqing Li.

**Data curation:** Ziqing Li.

**Formal analysis:** Ziqing Li, Tie Yuan.

**Funding acquisition:** Ziqing Li, Tie Yuan.

**Methodology:** Yu Zhang, Yuanping Wang.

**Project administration:** Yu Zhang.

## Supplementary Material

Supplemental Digital Content
