# Supplementary material for: Clinical efficacy and safety of nuanxin capsule for chronic heart failure: A systematic review and meta-analysis
Source: Medicine (Baltimore). 2018 Jul 6;97(27):e11339. doi: 10.1097/MD.0000000000011339 (PMC6076094; doi:10.1097/MD.0000000000011339)
Supplement: Supplemental Digital Content [file medi-97-e11339-s001.docx]

**Appendix A.**

***Search strategy used in PubMed database***

#1 nuanxin capsule

#2 Heart Failure OR Cardiac Failure OR Heart Decompensation OR Decompensation, Heart OR Heart Failure, Right-Sided OR Heart Failure, Right Sided OR Right-Sided Heart Failure OR Right Sided Heart Failure OR Myocardial Failure OR Congestive Heart Failure OR Heart Failure, Congestive OR Heart Failure, Left-Sided OR Heart Failure, Left Sided OR Left-Sided Heart Failure OR Left Sided Heart Failure

#3 Randomized controlled trial OR clinical study OR Clinical Trial OR Controlled study OR Controlled Trial OR Random*Control* study OR random* Control* Trial

#1 AND #2 AND #3
